# Antioxidative Activity of 1,3,5-Triazine Analogues Incorporating Aminobenzene Sulfonamide, Aminoalcohol/Phenol, Piperazine, Chalcone, or Stilbene Motifs

**DOI:** 10.3390/molecules25081787

**Published:** 2020-04-14

**Authors:** Eva Havránková, Nikola Čalkovská, Tereza Padrtová, Jozef Csöllei, Radka Opatřilová, Pavel Pazdera

**Affiliations:** 1 Department of Chemical Drugs, Faculty of Pharmacy, University of Veterinary and Pharmaceutical Sciences, 612 42 Brno, Czech Republic; F16222@vfu.cz (N.Č.); padrtovat@vfu.cz (T.P.); csolleij@vfu.cz (J.C.); opatrilovar@vfu.cz (R.O.); 2 Department of Chemistry, Faculty of Science, Centre for Syntheses at Sustainable Conditions and Their Management, Masaryk University, 625 00 Brno, Czech Republic; pazdera@chemi.muni.cz

**Keywords:** 1,3,5-triazine, 4-aminophenol, hydroxychalcone, hydroxystilbene, antioxidative activity, ABTS method

## Abstract

A series of 1,3,5-triazine analogues, incorporating aminobenzene sulfonamide, aminoalcohol/phenol, piperazine, chalcone, or stilbene structural motifs, were evaluated as potential antioxidants. The compounds were prepared by using step-by-step nucleophilic substitution of chlorine atoms in starting 2,4,6-trichloro-1,3,5-triazine. Reactions were catalyzed by Cu(I)-supported on a weakly acidic resin. The radical scavenging activity was determined in terms of %inhibition activity and EC_50_, using the ABTS method. Trolox and ascorbic acid (ASA) were used as standards. In the lowest concentration 1 × 10^−4^ M, the %inhibition activity values at 0 min were comparable with both standards at least for 10 compounds. After 60 min, compounds **5**, **6**, **13**, and **25** showed nearly twice %inhibition (73.44–87.09%) in comparison with the standards (Trolox = 41.49%; ASA = 31.07%). Values of EC_50_ at 60 min (17.16–27.78 μM) were 5 times lower for compounds **5**, **6**, **13**, and **25** than EC_50_ of both standards (trolox = 178.33 μM; ASA = 147.47 μM). Values of EC_50_ correlated with %inhibition activity. Based on these results, the presented 1,3,5-triazine analogues have a high potential in the treatment of illnesses caused or related to oxidative stress.

## 1. Introduction

Oxidative stress or damage is the imbalance between the capacity of antioxidative protection systems of the organism and the occurrence of reactive oxygen species and/or reactive nitrogen species [1,2]. Oxygen reactive species, relevant as free radicals, can be produced by metabolic pathways, UV irradiation, environmental pollutants, and others [1,2,3,4,5,6].

Damage of cells caused by oxidative stress is related to pathophysiology and pathogenesis of a wide range of diseases, i.e., Alzheimer’s, Huntington’s, and Parkinson’s diseases, inflammatory diseases, cardiovascular diseases, diabetes, and cancer [1,3,4,7,8,9].

Cytostatics, particularly drugs targeting DNA (e.g., doxorubicin and daunorubicin), highly induce the levels of oxidative stress [10,11,12]. This is one of the reasons why cytostatics possess chemotherapy-induced toxicity and a broad spectra of side effects [11,12,13]. Oxidative stress slows down the progression of the cell cycle, interferes with drug-induced apoptosis, and can diminish the effectiveness of the treatment. These processes are significant for cytostatics to exert their optimal cytotoxicity on tumor cells. Therefore, the administration of suitable antioxidants at specific dosages can enhance the potency of chemotherapy [11,12,14,15]. According to Singh et al., antioxidants do not interfere with the effect of the cytostatics; on the contrary, the enhanced anticancer activity is expected. Oxidation inhibitors also help to protect the normal healthy cells from the damage caused by free radicals, increase the therapeutic response of patients, and can help them to minimalize some of the chemotherapy side effects [11,16,17].

**Figure 1:**
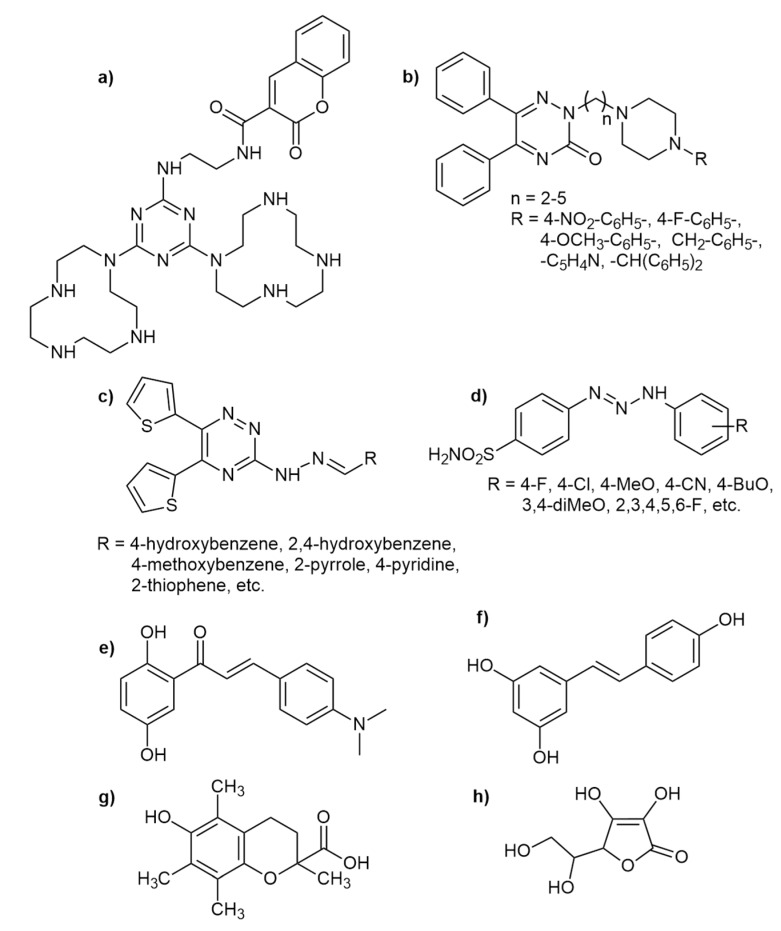
Examples of diversity of antioxidants with high activity: (**a**) 1,3,5-triazine bridged small molecule [3]; (**b**) biphenyl-3-oxo-1,2,4-triazine linked piperazines [26]; (**c**) arylidene hydrazine derivatives of substituted 1,2,4-triazine [27]; (**d**) substituted aminobenzene sulfonamides [28]; (**e**) chalcone derivative [29]; (**f**) resveratrol [4]; (**g**) trolox [4]; (**h**) ascorbic acid [4].

2,4,6-trichloro-1,3,5-triazine itself is a highly toxic and harmful substance. It can cause an allergic skin reaction, eye damage, and respiratory irritation [18,19]. Piperazine was used in the past as an anthelmintic by agonistic effect upon GABA receptors [20]. The antibacterial effect of aminobenzene sulphonamide was also proved. It slows down the metabolism of folic acid by competitive inhibition of enzymatic reactions involving *p*-aminobenzoic acid [21]. The combination of structures with biological activity (stilbenes, chalcones, piperazines, aminoalcohols/phenols, and aminobenzene sulfonamides) with a 1,3,5-triazine core (privileged structure) can lead to a significant increase in the antioxidative activity [22,23,24,25]. The molecular structure of compounds with radical scavenging activity can be very diverse. Recently, triazine derivatives with desirable activity were described. Gonzalez et al. proved the antioxidant activity of their 1,3,5-triazine bridged small molecules (Figure 1a) [3]. 5,6-Diphenyl-3-oxo-1,2,4-triazine linked piperazine analogues (Figure 1b) [26] or arylidene hydrazine derivatives of substituted 1,2,4-triazine scaffolds also exhibited attractive radical scavenging effects (Figure 1c) [27]. Substituted triazene benzene sulfonamides (Figure 1d), synthesized by Akocak et al., also showed significant antioxidative activity [28].

Promising ability to scavenge free radicals can be found even between chalcones. For example, Nasringhani et al. synthesized very effective chalcones containing a phenolic functional group (Figure 1e) [29].

Phenolic compounds can be considered as one of the largest groups of antioxidants with high activity [1,4,7,8,9,30].

The various stilbenoids can affect the formation of oxygen radicals in a cellular model. They might have an effect on the production of catalase, heme oxygenase-1, and glutathione peroxidase enzymes. Depending on their molecular structure, they can produce antioxidative but also pro-oxidative effects [31]. Resveratrol (stilbene analogue; see Figure 1f) is an example of phenolic antioxidant, which serves in medicine as an adjuvant treatment of cardiovascular diseases, metabolic syndrome, type 2 diabetes, and inflammatory diseases [4].

Antioxidants possess great potential as drugs suitable for the treatment of various diseases related to oxidative stress. In particular, the use of compounds containing phenol structural moiety in the treatment of Alzheimer’s disease [32,33,34,35,36], Parkinson´s disease [37,38,39], Huntington´s disease [40,41], and cancer [42,43,44,45] have been extensively studied in the last two years. Their structural diversity provides many possibilities for the design and synthesis of new, highly effective agents.

In this paper, a series of 2,4,6-trichloro-1,3,5-triazine analogues, incorporating aminobenzene sulfonamide, aminoalcohol, piperazine, chalcone, or stilbene structural motifs, were evaluated as potential antioxidants.

## 2. Results and Discussion

### 2.1. Chemistry

**Scheme 1:**
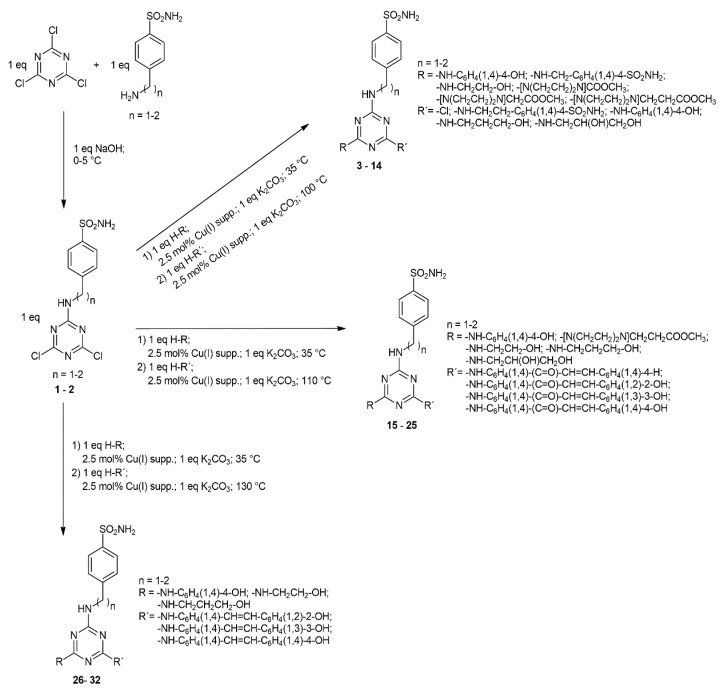
General synthetic scheme of target compounds. Compounds (**3**–**14**) contain aminoalcohol/phenol or piperazine structural motifs; compounds (**15**–**25**) contain chalcone structural motif; compounds (**26**–**32**) contain stilbene structural motif.

Starting monosubstituted dichlorotriazinyl (**1**–**2**) benzene sulfonamides were prepared as reported by Garaj et al. [46]. Target compounds (**3**–**32**) were synthesized according to the methodology published by our team [47] by step-by-step nucleophilic substitution of chlorine atoms of 2,4,6-trichloro-1,3,5-triazine. The appropriate starting compound reacted with a nucleophile in the presence of anhydrous potassium carbonate in a molar ratio of 1:1:1. Reactions were catalyzed by Cu(I)-supported on a weakly acidic resin. The substitution of a first, second, or third chlorine atom was controlled by the temperature mode (Scheme 1). The %yields of reactions depend very strongly on the nucleophilicity and size of appropriate nucleophiles. Substitution of the third chlorine atom in disubstituted intermediates with chalcones provides much better yields than for reactions where stilbenes act as nucleophiles. Substituents present on the 1,3,5-triazine core also play an important role. With the increasing number of CH_2_ in the aminobenzene sulfonamide, structural moiety decreases the electron-withdrawing character of the sulfonamide functional group, which leads to worse reactivity of disubstituted intermediates.

### 2.2. Antioxidant Evaluation

In this paper, a series of 2,4,6-trichloro-1,3,5-triazine analogues, incorporating aminobenzene sulfonamide, aminoalcohol/phenol, piperazine, chalcone, or stilbene structural motifs, were evaluated as potential antioxidants. These analogues were primarily designed as inhibitors of hCA IX (isozyme of human carbonic anhydrase), and the activity of this enzyme is correlated with tumor growth. Since the synthesized compounds contain one or more structural motifs that already have demonstrated antioxidant properties (Chapter 1), the screening of their antioxidant activity was done. Because our compounds contain either 4-aminophenol or hydroxystilbene or hydroxychalcone structural moieties, we assumed the mechanism of action to be very similar to resveratrol and analogous compounds. Currently, the ABTS method is one of the most widely used for the determination of the antioxidative properties of phenols [30]. For this reason, the antioxidant activity of tested compounds was determined by the ABTS assay. The results were compared to commercially available standards for trolox and ascorbic acid.

We were pleased to find that almost half of the tested compounds exhibited excellent antioxidative activity. The results of the (cation)radical scavenging activity are shown in Table 1 (%inhibition activity and values of EC_50_, respectively).

Over twenty compounds fully captured the cation radical ABTS**^.^**^+^ after a few seconds (time 0 min) at the highest used concentration of 1 × 10^−2^ M. At the lowest concentration of 1 × 10^−4^ M at 0 min, for ten compounds, %inhibition of the cation radical ABTS**^.^**^+^ was comparable or better than both used standards (trolox and ascorbic acid). Furthermore, compounds **5**, **6**, **13**, **24**, and **25** showed after 60 min almost two times higher %inhibition activity (73.44–87.09%) in comparison to standards for trolox (41.09%) and ascorbic acid (31.07%).

The values of EC_50_ were obtained as described in the experimental section, and they correlated with the results of the determination of the %inhibition of cation radical ABTS**^.^**^+^. From the results presented in Table 1, it is clear that EC50 values of the most active compounds after 60 min (EC50 = 17.16–27.78 μM) were nearly ten times lower than that of both used standards (trolox EC50 = 178.33; ascorbic acid EC_50_ = 147.47). The structure of the three compounds with the highest antioxidant activity (the lowest values of EC_50_) is very diverse: compound **6** is a disubstituted analogue containing aminobenzene sulfonamide and aminophenol structural motif; compound **22** contains aminobenzene sulfonamide, aminoethanol, and 3-hydroxy-aminochalcone structural fragments; and compound **25** contains aminobenzene sulfonamide, piperazine, and 4-hydroxy-aminochalcone structural motifs. All three compounds consist of the common structural fragment phenolic hydroxyl group, which is probably responsible for their antioxidative activity. On the other hand, compounds that contain the only combination of aminobenzene sulfonamide substituents and/or substituents without a phenolic hydroxyl group had none or moderate antioxidant activity.

From the results, some general statements can be made. Compounds that contain the phenolic fragment in their structure had the highest values of %inhibition activity. The hydroxy group can inhibit the cation radical ABTS**^.^**^+^, probably by the single-electron transfer mechanism (SET). The resulting cation radical formed after scavenging is stabilized by the delocalization of the electron across the molecule. The presence of the oxo group (C=O) and the unsaturated double bond in the chalcone structural motif enhanced the antioxidative properties of the phenolic hydroxy group as compared to a simple amino phenol substituent. This is caused by a very high electron delocalization of the formed cation radical, which is supported by the electron-withdrawing character of the carbonyl group. In fact, the compounds containing the hydroxychalcone moieties are, in general, the most active of tested compounds. On the other hand, the compounds incorporating the stilbene structural motif exhibit lower %inhibition of the cation radical ABTS**^.^**^+^. The reason is less effective conjugation of the cation radical in comparison with the chalcone derivatives. In general, a higher level of electron delocalization of formed cation radical increases the energy of HOMO, which means the better oxidation-reduction potential of the compound and, therefore, the better oxidative activity.

To summarize, tested compounds were demonstrated as valuable potential as antioxidants useful in the treatment of diseases caused by oxidative stress. Besides, the antioxidant effect is a great benefit for compounds that are excellent inhibitors of hCA IX (compound **5**) [48] since oxidative stress is associated with tumor development and progression in several malignancies. In addition, the antioxidative properties may have a beneficial effect in suppressing the side effects associated with the treatment using some classic cytostatic drugs [11,49].

## 3. Conclusions

In this paper, a series of 1,3,5-triazine derivatives, incorporating aminobenzene sulfonamide, aminoalcohol/phenol, piperazine, chalcone, or stilbene structural motifs, were evaluated as potential antioxidants. At the lowest concentration 1x10^−4^ M at 0 min, %inhibition of the cation radical ABTS**^.^**^+^ was comparable or better than both used standards for ten compounds. Furthermore, after 60 min, compounds **5**, **6**, **13**, and **25** showed nearly twice %inhibition (73.44–87.09%) in comparison with standards (trolox = 41.49%; ASA = 31.07%). For compounds **5**, **6**, **13**, and **25**, values of EC_50_ at 60 min (17.16–27.78 μM) were 5-fold lower than EC_50_ of both standards (trolox = 178.33 μM; ASA = 147.47 μM). From these results, more than half of the tested compounds showed great potential as antioxidants useful in the treatment of diseases caused by oxidative stress.

## 4. Experimental Section

### 4.1. General Information

All reagents were purchased from commercial suppliers (Sigma-Aldrich, Darmstadt, Germany) and used as supplied without further purification. The used catalytic system of Cu(I)-supported on a weakly acidic resin is commercially available at www.entwickchemicals.com.

All the reactions were monitored by TLC performed on precoated silica gel 60 F254 plates (Merck, Darmstadt, Germany). For compounds **3**–**14**, the methanol was used as an eluent; UV light (254 and 356 nm) and ninhydrin reagent were used for the detection of spots at 180 °C. For compounds **15**–**32**, methanol:dichloromethane = 1:1 was used as an eluent; UV light (254 and 356 nm), iodine, and ninhydrin reagent were used for the detection of spots at 180 °C. NMR spectra were recorded on DRX 500 Avance (Bruker Biospin, Billerica, MA, USA) spectrometer using TMS as an internal standard. The FTIR spectra were obtained on an Alpha II FTIR Spectrometer (Bruker, Billerica, MA, USA) equipped with the ATR module. Melting points (uncorrected) were recorded on Kofler’s block Boetius Rapido PHMK 79/2106 (Wagetechnik, Dresden, Germany) with temperature gradient 4 °C.min^−1^.

All spectral data of known compounds (**3**–**14**) agreed with those previously reported [48].

### 4.2. General Synthetic Procedures

Starting monosubstituted dichlorotriazinyl benzene sulfonamides were prepared according to the literature [46].

#### 4.2.1. General Method for Synthesis of Trisubstituted Derivatives of 1,3,5-Triazine Containing Aminoalcohol/Phenol or Piperazine Structural Motifs (**3**–**14**)

Compounds **3**–**14** were prepared according to the methodology published in [48]. Reactions were upgraded by using the catalytic system of Cu(I)-supported on a weakly acidic resin.

All spectral data of known compounds (**3**–**14**) agreed with those previously reported [48].

#### 4.2.2. General Method for Synthesis of Trisubstituted Derivatives of 1,3,5-Triazine Containing Chalcone Structural Motif (**15**–**25**)

Step 1: Starting dichlorotriazinyl benzene sulfonamide (1 mmol) was dissolved in 10 mL of DMF. One mmol of solid anhydrous potassium carbonate was added in small portions, and the mixture was stirred for 10 min. Then, one mmol of the appropriate nucleophile was added portion-wise. Finally, 2.5% mol of supported Cu(I) ions were added into the reaction mixture. The reaction mass was stirred at 35 °C until the maximum conversion of starting reactants was achieved (monitored by TLC). After the completion of a reaction, the catalyst and salt were filtered off. Crushed ice was then added into the solution, and the formed precipitate was collected by filtration. The crude product was dissolved in hot acetone and precipitated by the addition of isopropyl alcohol.

Step 2: Appropriate chalcone (1 mmol) was dissolved in 15 mL of DMF. One mmol of solid anhydrous potassium carbonate was added in small portions, and the mixture was stirred for 15 min. Then, one mmol of the appropriate nucleophile (a disubstituted derivative of cyanuric chloride) was added portion-wise. Finally, 2.5% mol of supported Cu(I) ions were added into the reaction mixture. The reaction was stirred at 110 °C until the maximum conversion of starting reactants was achieved (monitored by TLC). After the completion of a reaction, the catalyst and salt were filtered off. The filtrate was concentrated to 1/5 of the original volume by rotary vacuum evaporator. The pure product was obtained by the addition of the mixture of isopropyl alcohol:diethyl ether (1:10) and filtered off. The obtained products were stored under the inert atmosphere (argon).

#### 4.2.3. General Method for Synthesis of Trisubstituted Derivatives of 1,3,5-Triazine Containing Stilbene Structural Motif (**26**–**32**)

Step 1: Starting dichlorotriazinyl benzene sulfonamide (1 mmol) was dissolved in 10 mL of DMF. One mmol of solid anhydrous potassium carbonate was added in small portions, and the mixture was stirred for 10 min. Next, one mmol of the appropriate nucleophile was added portion-wise. Finally, 2.5% mol of supported Cu(I) ions were added into the reaction mixture. The reaction mass was stirred at 35 °C until the maximum conversion of starting reactants was achieved (monitored by TLC). After the completion of a reaction, the catalyst and salt were filtered off. Crushed ice was added into the solution, and the formed precipitate was collected by filtration. The crude product was dissolved in hot acetone and precipitated by the addition of isopropyl alcohol.

Step 2: Appropriate stilbene (1 mmol) was dissolved in 15 mL of DMF. One mmol of solid anhydrous potassium carbonate was added in small portions, and the mixture was stirred for 15 min. Then, one mmol of the appropriate nucleophile (a disubstituted derivative of cyanuric chloride) was added portion-wise. Finally, 2.5% mol of supported Cu(I) ions were added to the reaction mixture. The reaction mass was stirred at 130 °C until the maximum conversion of the starting reactants was achieved (monitored by TLC). After the completion of the reaction, the catalyst and salt were filtered off. The filtrate was concentrated to 1/10 of the original volume by a rotary vacuum evaporator, and 15 mL of isopropyl alcohol was added. The mixture was cooled at 0–5 °C overnight. The obtained pure product was filtered off. The other portion of pure product was obtained as follows: The filtrate was treated with a few drops of diethyl ether, and then the solvent was evaporated entirely by a rotary vacuum evaporator. Then, 2 mL of cold water was poured, and the crystals were formed. The mixture was cooled to 0–5 °C (in the fridge) for 72 h and then filtered.

#### 4.2.4. Characterization of New Compounds

*4-[({4-Chloro-6-[(2-hydroxyethyl)amino]-1,3,5-triazin-2-yl}amino)methyl]benzenesulfonamide* (**3**): 60.9%; white solid; mp 223–224 °C; ^1^H-NMR (500 MHz, DMSO-d_6_) δ ppm 7.77 (2H, d, *J* = 8.1 Hz, CH), 7.45 (2H, d, *J* = 8.1 Hz, CH), 4.8 (5H, s, OH, NH, NH_2_), 4.51 (2H, s, NH-CH_2_), 3.48-3.42 (2H, m, CH_2_-OH), 3.29-3.27 (2H, m, NH-CH_2_); ^13^C-NMR (125 MHz, DMSO-*d*_6_) δ ppm 168.9, 168.0, 145.7, 142.0, 128.0, 126.8, 60.7, 44.5, 43.4; IR ν_max_ (cm^−1^) 3403 (OH, NH, NH_2_), 3245, 2945 (CH_aliph_), 1633 (C=Carom), 1486, 1400, 1155 (SO_2_NH_2_).

*4-[2-({4-Chloro-6-[(2-hydroxyethyl)amino]-1,3,5-triazin-2-yl}amino)ethyl]benzenesulfonamide* (**4**): 89.7%; white solid; mp 127–128 °C; ^1^H-NMR (500 MHz, DMSO-d_6_) δ ppm 7.73 (2H, d, *J* = 8.1 Hz, CH), 7.41 (2H, d, *J* = 8.1 Hz, CH), 5.8 (5H, s, OH, NH, NH_2_), 3.53-3.51 (2H, m, CH_2_-OH), 3.49-3.47 (2H, m, NH-CH_2_), 3.37-3.35 (2H, m, NH-CH_2_), 3.29-3.27 (2H, m, CH_2_); ^13^C-NMR (125 MHz, DMSO-*d*_6_) δ ppm 168.5, 168.1, 167.8, 143.9, 142.9, 129.6, 126.0, 59.8, 43.5, 43.4, 34.6; IR ν_max_ (cm^−1^) 3327 (OH, NH, NH_2_), 3248, 2946 (CH_aliph_), 1636 (C=C), 1452, 1439, 1154 (SO_2_NH_2_).

*(E)-4-[({4-[(4-Cinnamoylphenyl)amino]-6-[(3-hydroxypropyl)amino]-1,3,5-triazin-2-yl}amino)methyl]benzenesulfonamide* (**15**): 89.7%; ochre solid; mp 168–171 °C; ^1^H-NMR (500 MHz, DMSO-d_6_) δ ppm 7.97 (2H, d, *J* = 8.1 Hz, CH), 7.84 (2H, d, *J* = 8.1 Hz, CH), 7.78 (1H, d, *J* = 15.5 Hz, CH=CH-CO), 7.60 (2H, d, *J* = 8.1 Hz, CH), 7.55 (1H, d, *J* = 15.5 Hz, CH=CH-CO), 7.50 (2H, d, *J* = 8.1 Hz, CH), 7.40 (2H, d, *J* = 8.1 Hz, CH), 7.31-7.25 (3H, m, CH), 6.35 (6H, s, OH, NH, NH_2_), 4.62-4.59 (2H, m, CH_2_), 3.62-3.59 (2H, m, CH_2_-OH), 3.53-3.48 (2H, m, NH-CH_2_), 2.05-2.01 (2H, m, CH_2_); ^13^C-NMR (125 MHz, DMSO-*d*_6_) δ ppm 189.4, 168.6, 165.9, 165.8, 145.7, 144.3, 142.0, 141.8, 141.5, 136.8, 129.9, 129.0, 128.0, 126.8, 124.6, 123.9, 122.0, 119.0, 57.9, 44.4, 38.6, 22.7; IR ν_max_ (cm^−1^) 3343, 3231 (OH, NH, NH_2_), 2930 (CH_aliph_), 1698, 1662, 1635 (C=C, C=N, C=O), 1574, 1338, 1177, (SO_2_NH_2_), 1034 (COC).

*(E)-4-[2-({4-[(4-Cinnamoylphenyl)amino]-6-[(3-hydroxypropyl)amino]-1,3,5-triazin-2-yl}amino)ethyl]benzenesulfonamide* (**16**): 81.1%; yellow solid; mp 136–138 °C; ^1^H-NMR (500 MHz, DMSO-d_6_) δ ppm 7.96 (2H, d, *J* = 8.1 Hz, CH), 7.83 (2H, d, *J* = 8.1 Hz, CH), 7.75 (1H, d, *J* = 15.5 Hz, CH=CH-CO), 7.57 (2H, d, *J* = 8.1 Hz, CH), 7.55 (1H, d, *J* = 15.5 Hz, CH=CH-CO), 7.51 (2H, d, *J* = 8.1 Hz, CH), 7.36 (2H, d, *J* = 8.1 Hz, CH), 7.22-7.19 (3H, m, CH), 6.35 (6H, s, OH, NH, NH_2_), 4.61-4.59 (2H, m, CH_2_), 3.63-3.58 (2H, m, CH_2_-OH), 3.54-3.47 (2H, m, NH-CH_2_), 2.88-2.84 (2H, m, CH_2_), 2.11-2.07 (2H, m, CH_2_); ^13^C-NMR (125 MHz, DMSO-*d*_6_) δ ppm 189.2 (C=O), 168.5, 165.7, 165.7, 145. 6, 144.1, 142.1, 141.5, 141.2, 136.7, 129.8, 128.9, 127.8, 126.6, 124.3, 123.7, 122.1, 118.8, 57.2, 44.7, 38.3, 32.4, 22.2; IR ν_max_ (cm^−1^) 3335, 3229 (OH, NH, NH_2_), 2942 (CH_aliph_), 1698, 1681, 1651, 1642 (C=C, C=N, C=O), 1592, 1337, 1157 (SO_2_NH_2_), 1028 (COC).

*(E)-4-[({4-({4-[3-(3-Hydroxyphenyl)acryloyl]phenyl}amino)-6-[(3-hydroxypropyl)amino]-1,3,5-triazin-2-yl}amino)methyl]benzenesulfonamide* (**17**): 78.3%; brown solid; mp 346–348 °C; ^1^H-NMR (500 MHz, DMSO-d_6_) δ ppm 7.96 (2H, d, J = 8.1 Hz, CH), 7.80 (2H, d, J = 8.1 Hz, CH), 7.76 (1H, d, J = 15.5 Hz, CH=CH-CO), 7.58 (2H, d, J = 8.1 Hz, CH), 7.53 (1H, d, J = 15.5 Hz, CH=CH-CO), 7.28 (2H, d, J = 8.1 Hz, CH), 6.87 (2H, d, J = 8.1 Hz, CH), 6.74 (7H, s, OH, NH, NH_2_), 4.58-4.53 (2H, m, CH_2_), 3.60-3.57 (2H, m, CH_2_-OH), 3.41-3.39 (2H, m, NH-CH_2_), 1.91-1.90 (2H, m, CH_2_); ^13^C-NMR (125 MHz, DMSO-d_6_) δ ppm 189.3, 168.4, 165.7, 164.9, 146.7, 144.2, 143.8, 141.8, 141.4, 139.9, 138.5, 130.5, 128.2, 126.4, 126.1, 119.9, 119.2, 114.6, 57.8, 44.2, 38.5, 32.1; IR ν_max_ (cm^−1^) 3331, 3227 (OH, NH, NH_2_), 2931 (CH_aliph_), 1685, 1636, 1594 (C=C, C=N, C=O), 1570, 1342, 1157, (SO_2_NH_2_), 1086 (C-OH), 1022 (COC).

*(E)-4-[({4-({4-[3-(2-Hydroxyphenyl)acryloyl]phenyl}amino)-6-[(3-hydroxypropyl)amino]-1,3,5-triazin-2-yl}amino)methyl]benzenesulfonamide* (**18**): 75.3%; brown solid; mp 178–179 °C; ^1^H-NMR (500 MHz, DMSO-d_6_) δ ppm 7.96 (2H, d, J = 8.1 Hz, CH), 7.85 (2H, d, J = 8.1 Hz, CH), 7.71 (1H, d, J = 15.5 Hz, CH=CH-CO), 7.58 (2H, d, J = 8.1 Hz, CH), 7.49 (1H, d, J = 15.5 Hz, CH=CH-CO), 7.28 (2H, d, J = 8.1 Hz, CH), 6.92 (2H, d, J = 8.1 Hz, CH), 6.59 (7H, s, OH, NH, NH_2_), 4.59-4.57 (2H, m, CH_2_), 3.62-3.59 (2H, m, CH_2_-OH), 3.42-3.39 (2H, m, NH-CH_2_), 2.05-2.01 (2H, m, CH_2_); ^13^C-NMR (125 MHz, DMSO-d_6_) δ ppm 188.7, 168.5, 166.1, 165.9, 159.6, 144.4, 141.5, 139.1, 141.9, 139.7, 139.0, 129.8, 128.9, 127.7, 124.8, 120.3, 118.7, 115.2, 57.8, 44.1, 38.6, 32.1; IR ν_max_ (cm^−1^) 3353, 3226 (OH, NH, NH_2_), 2929 (CH_aliph_), 1659, 1652, 1623, 1588 (C=C, C=N, C=O), 1334, 1159 (SO_2_NH_2_), 1087 (C-OH), 1019 (COC).

*(E)-4-[2-({4-({4-[3-(2-Hydroxyphenyl)acryloyl]phenyl}amino)-6-[(3-hydroxypropyl)amino]-1,3,5-triazin-2-yl}amino)ethyl]benzenesulfonamide* (**19**): 48.3%; brown solid; mp 229–231 °C; ^1^H-NMR (500 MHz, DMSO-d_6_) δ ppm 7.99 (2H, d, J = 8.1 Hz, CH), 7.87 (2H, d, J = 8.1 Hz, CH), 7.81 (1H, d, J = 15.5 Hz, CH=CH-CO), 7.62 (2H, d, J = 8.1 Hz, CH), 7.51 (1H, d, J = 15.5 Hz, CH=CH-CO), 7.36 (2H, d, J = 8.1 Hz, CH), 7.25 (2H, d, J = 8.1 Hz, CH), 6.97 (7H, s, OH, NH, NH_2_), 4.21-4.17 (2H, m, CH_2_), 3.69-3.66 (2H, m, CH_2_-OH), 3.45-3.42 (2H, m, NH-CH_2_), 2.99-2.95 (2H, m, CH_2_), 2.13-2.09 (2H, m, CH_2_); ^13^C-NMR (125 MHz, DMSO-d_6_) δ ppm 189.2, 168.9, 166.7, 166.1, 159.0, 144.6, 143.9, 141.4, 139.9, 140.6, 138.9, 130.6, 129.2, 128.5, 125.87, 120.7, 118.4, 116.2, 57.6, 43.9, 38.7, 33.9, 32.1; IR ν_max_ (cm^−1^) 3336, 3308 (OH, NH, NH_2_), 1933 (CH_aliph_, CH_2_), 1698, 1682, 1668, 1662, 1651, 1622 (C=C, C=N, C=O), 1574, 1346, 1176, (SO_2_NH_2_), 1079 (C-OH), 1035 (COC).

*(E)-4-[({4-[(2-Hydroxyethyl)amino]-6-({4-[3-(2-hydroxyphenyl)acryloyl]phenyl}amino)-1,3,5-triazin-2-yl}amino)methyl]benzenesulfonamide* (**20**): 68.7%; brown solid; mp 246–248 °C; ^1^H-NMR (500 MHz, DMSO-d_6_) δ ppm 7.93 (2H, d, J = 8.1 Hz, CH), 7.81 (2H, d, J = 8.1 Hz, CH), 7.72 (1H, d, J = 15.5 Hz, CH=CH-CO), 7.58 (2H, d, J = 8.1 Hz, CH), 7.51 (1H, d, J = 15.5 Hz, CH=CH-CO), 7.28 (2H, d, J = 8.1 Hz, CH), 6.92 (2H, d, J = 8.1 Hz, CH), 6.71 (7H, s, OH, NH, NH_2_), 4.58-4.56 (2H, m, CH_2_), 3.57-3.53 (2H, m, CH_2_-OH), 3.36-3.32 (2H, m, NH-CH_2_); ^13^C-NMR (125 MHz, DMSO-d_6_) δ ppm 189.6, 168.5, 166.0, 165.2, 146.9, 144.7, 144.4, 142.2, 141.9, 141.3, 138.3, 130.6, 128.3, 126.7, 126.2, 119.8, 118.7, 114.6, 58.8, 44.7, 42.1; IR ν_max_ (cm^−1^) 3331, 3224 (OH, NH, NH_2_), 2932 (CH_aliph_), 1678, 1640, 1586 (C=C, C=N, C=O), 1556, 1342, 1156, (SO_2_NH_2_), 1086 (C-OH), 1027 (COC).

*(E)-4-[2-({4-[(2-Hydroxyethyl)amino]-6-({4-[3-(2-hydroxyphenyl)acryloyl]phenyl}amino)-1,3,5-triazin-2-yl}amino)ethyl]benzenesulfonamide* (**21**): 70.8%; yellow solid; mp 261–262 °C; ^1^H-NMR (500 MHz, DMSO-d_6_) δ ppm 7.91 (2H, d, J = 8.1 Hz, CH), 7.82 (2H, d, J = 8.1 Hz, CH), 7.74 (1H, d, J = 15.5 Hz, CH=CH-CO), 7.61 (2H, d, J = 8.1 Hz, CH), 7.57 (1H, d, J = 15.5 Hz, CH=CH-CO), 7.32 (2H, d, J = 8.1 Hz, CH), 6.89 (2H, d, J = 8.1 Hz, CH), 6.70 (7H, s, OH, NH, NH_2_), 4.57-4.56 (2H, m, CH_2_), 3.55-3.51 (2H, m, CH_2_-OH), 3.33-3.29 (2H, m, NH-CH_2_); ^13^C-NMR (125 MHz, DMSO-d_6_) δ ppm 189.7, 168.2, 166.3, 165.4, 147.0, 144.8, 144.6, 142.3, 141.7, 141.2, 138.1, 130.4, 128.6, 126.9, 126.5, 119.7, 118.6, 114.7, 58.6, 44.3, 41.9; IR ν_max_ (cm^−1^) 3341, 3224 (OH, NH, NH_2_), 2935 (CH_aliph_), 1698, 1661, 1654, 1633 (C=C, C=N, C=O), 1575, 1345, 1131, (SO_2_NH_2_), 1078 (C-OH), 1034 (COC)

*(E)-4-[2-({4-[(2-Hydroxyethyl)amino]-6-({4-[3-(3-hydroxyphenyl)acryloyl]phenyl}amino)-1,3,5-triazin-2-yl}amino)ethyl]benzenesulfonamide* (**22**): 77.7%; brown solid; mp 256–258 °C; ^1^H-NMR (500 MHz, DMSO-d_6_) δ ppm 7.91 (2H, d, J = 8.1 Hz, CH), 7.80 (2H, d, J = 8.1 Hz, CH), 7.71 (1H, d, J = 15.5 Hz, CH=CH-CO), 7.56 (2H, d, J = 8.1 Hz, CH), 7.48 (1H, d, J = 15.5 Hz, CH=CH-CO), 7.26 (2H, d, J = 8.1 Hz, CH), 6.91 (2H, d, J = 8.1 Hz, CH), 6.69 (7H, s, OH, NH, NH_2_), 4.56-4.52 (2H, m, CH_2_), 3.55-3.53 (2H, m, CH_2_-OH), 3.34-3.32 (2H, m, NH-CH_2_), 2.93-2.91 (2H, m, CH_2_); ^13^C-NMR (125 MHz, DMSO-d_6_) δ ppm 189.4, 168.6, 166.3, 165.6, 146.7, 144.5, 144.1, 142.3, 141.8, 141.2, 138.2, 130.4, 128.2, 126.6, 125.9, 119.7, 118.6, 114.2, 58.8, 44.5, 41.9, 36.6; IR ν_max_ (cm^−1^) 3336, 3222 (OH, NH, NH_2_), 2930 (CH_aliph_), 1698, 1660, 1654, 1635 (C=C, C=N, C=O), 1583, 1345, 1155, (SO_2_NH_2_), 1076 (C-OH), 1034 (COC).

*(E)-4-[2-({4-[(2,3-Dihydroxypropyl)amino]-6-({4-[3-(2-hydroxyphenyl)acryloyl]phenyl}amino)-1,3,5-triazin-2-yl}amino)ethyl]benzenesulfonamide* (**23**): 77.6%; brown solid; mp 276–278 °C; ^1^H-NMR (500 MHz, DMSO-d_6_) δ ppm 7.94 (2H, d, J = 8.1 Hz, CH), 7.89 (2H, d, J = 8.1 Hz, CH), 7.79 (1H, d, J = 15.5 Hz, CH=CH-CO), 7.71 (2H, d, J = 8.1 Hz, CH), 7.42 (2H, d, J = 8.1 Hz, CH), 7.34 (1H, d, J = 15.5 Hz, CH=CH-CO), 6.91 (2H, d, J = 8.1 Hz, CH), 6.67 (2H, d, J = 8.1 Hz, CH), 6.16 (8H, s, OH, NH, NH_2_), 4.57-4.55 (1H, m, CH-OH), 3.81-3.79 (4H, m, CH_2_), 3.20-3.17 (2H, m, NH-CH_2_), 2.89-2.86 (2H, m, CH_2_); ^13^C-NMR (125 MHz, DMSO-d_6_) δ ppm 185.4, 170.4, 167.6, 167.1, 154.1, 144.3, 144.0, 142.6, 142.1, 141.1, 139.7, 131.4, 129.4, 129.3, 126.1, 126.0, 125.8, 113.4, 70.8, 62.3, 44.3, 42.4, 36.5; IR ν_max_ (cm^−1^) 3351, 3219 (OH, NH, NH_2_), 2927 (CH_aliph_), 1669, 1654, 1625, 1592 (C=C, C=N, C=O), 1558, 1329, 1151 (SO_2_NH_2_), 1083 (C-OH), 1038 (COC).

*(E)-4-[2-({4-({4-[3-(2-Hydroxyphenyl)acryloyl]phenyl}amino)-6-[(4-hydroxyphenyl)amino]-1,3,5-triazin-2-yl}amino)ethyl]benzenesulfonamide* (**24**): 80.3%; brown solid; mp 198–199 °C; ^1^H-NMR (500 MHz, DMSO-d_6_) δ ppm 7.97 (2H, d, J = 8.1 Hz, CH), 7.86 (2H, d, J = 8.1 Hz, CH), 7.68 (1H, d, J = 15.5 Hz, CH=CH-CO), 7.55 (2H, d, J = 8.1 Hz, CH), 7.47 (1H, d, J = 15.5 Hz, CH=CH-CO), 7.34 (2H, d, J = 8.1 Hz, CH), 7.09 (2H, d, J = 8.1 Hz, CH), 6.99 (7H, s, OH, NH, NH_2_), 6.92 (2H, d, J = 8.1 Hz, CH), 6.85 (2H, d, J = 8.1 Hz, CH), 3.76-3.74 (2H, m, CH_2_), 2.95-2.91 (2H, m, CH_2_); ^13^C-NMR (125 MHz, DMSO-d_6_) δ ppm 188.9, 166.9, 166.7, 166.5, 159.7, 155.2, 143.9, 141.2, 139.9, 135.4, 135.0, 132.4, 132.1, 129.1, 128.1, 125.6, 124.8, 123.4, 121.1, 119.8, 117.3, 115.6, 43.3, 36.2; IR ν_max_ (cm^−1^) 3349, 3228 (OH, NH, NH_2_), 2932 (CH_aliph_), 1698, 1659, 1655, 1635, 1582 (C=C, C=N, C=O), 1543, 1327, 1156 (SO_2_NH_2_), 1090 (C-OH), 1011 (COC).

*(E)-Methyl 3-(4-{4-({4-[3-(4-hydroxyphenyl)acryloyl]phenyl}amino)-6-[(4-sulfamoyl-phenethyl)amino] -1,3,5-triazin-2-yl}piperazin-1-yl)propanoate* (**25**): 78.1%; orange solid; mp 101–103 °C; ^1^H-NMR (500 MHz, DMSO-d_6_) δ ppm 7.97 (2H, d, J = 8.1 Hz, CH), 7.94 (2H, d, J = 8.1 Hz, CH), 7.8 (1H, d, J = 15.5 Hz, CH=CH-CO), 7.42 (1H, d, J = 15.5 Hz, CH=CH-CO), 7.31 (2H, d, J = 8.1 Hz, CH), 7.28 (2H, d, J = 8.1 Hz, CH), 6.99 (2H, d, J = 8.1 Hz, CH), 6.68 (2H, d, J = 8.1 Hz, CH), 3.91-3.86 (4H, m, CH_2_), 3.74-3.71 (2H, m, NH-CH_2_), 3.65 (3H, s, CH_3_), 2.99-2.87 (4H, m, CH_2_), 2.76-2.74 (2H, m, CH_2_), 2.59 (2H, t, J=7.8 Hz, N-CH_2_), 2.11 (2H, t, J=7.8 Hz, CH_2_-COO); ^13^C-NMR (125 MHz, DMSO-d_6_) δ ppm 186.7, 171.9, 167.8, 167.1, 162.6, 159.9, 158.4, 154.2, 150.9, 143.8, 142.7, 144.3, 119.2, 131.3, 130.6, 126.8, 124.9, 116.3, 113.1, 66.4, 51.3, 51.1, 44.3, 43.7, 35.1, 31.6; IR ν_max_ (cm^−1^) 3332, 3218 (OH, NH, NH_2_), 2950 (CH_aliph_) 1698, 1683, 1669, 1651, 1646, 1630, 1588 (C=C, C=N, COO), 1349 (SO_2_NH_2_), 1227 (OH), 1162 (SO_2_NH_2_), 1105, 1028 (COC).

*(E)-4-{[(4-[(3-hydroxypropyl)amino]-6-{[4-(3-hydroxystyryl)phenyl]amino}-1,3,5-triazin-2-yl)amino]methyl}benzenesulfonamide* (**26**): 42.0%; ochre solid; mp 102–103 °C; ^1^H-NMR (500 MHz, DMSO-d_6_) δ ppm 9.07 (4H, br s, NH, NH_2_), 7.78 (2H, d, *J* = 7.8 Hz, CH), 7.74 (2H, d, *J* = 7.8 Hz, CH), 7.71-7.70 (1H, m, CH), 7.69-7.65 (3H, m, CH), 7.50 (2H, d, *J* = 8.5 Hz, CH), 7.43 (2H, d, *J* = 8.5 Hz, CH), 6.71 (3H, br s, OH, NH, NH_2_), 6.67-6.64 (2H, m, CH=CH), 4.65 (2H, s, NH-CH_2_), 3.63-3.61 (2H, m, CH_2_-OH), 3.47-3.44 (2H, m, NH-CH_2_), 2.32-2.27 (2H, m, NH-CH_2_CH_2_); ^13^C-NMR (125 MHz, DMSO-*d*_6_) δ ppm 166.2, 166.1, 166.0, 161.1, 144.8, 144.4, 139.8, 139.4, 139.1, 135.2, 132.6, 131.5, 129.7, 129.5, 129.1, 128.6, 127.4, 127.3, 126.8, 57.3, 43.9, 38.7, 32.3; IR ν_max_ (cm^−1^) 3365, 3224 (OH, NH, NH_2_), 3071, 3052 (CH_arom_), 2928, 2916 (CH_aliph_), 1686 (CH=CH), 1605, 1590, 1575 (C=C, C=N), 1334 (SO_2_NH_2_), 1233 (C-OH_phenol_), 1155 (SO_2_NH_2_), 1024 (C-OH_aliph_).

*(E)-4-{2-[(4-[(3-hydroxypropyl)amino]-6-{[4-(3-hydroxystyryl)phenyl]amino}-1,3,5-triazin-2-yl)amino]ethyl}benzenesulfonamide* (**27**): 27.0%; ochre solid; mp 158–160 °C; ^1^H-NMR (500 MHz, DMSO-d_6_) δ ppm 9.11 (4H, br s, NH, NH_2_), 7.80 (2H, d, *J* = 7.8 Hz, CH), 7.76 (2H, d, *J* = 7.8 Hz, CH), 7.76-7.72 (1H, m, CH), 7.69-7.65 (3H, m, CH), 7.52 (2H, d, *J* = 8.5 Hz, CH), 7.48 (2H, d, *J* = 8.5 Hz, CH), 6.98-6.95 (2H, m, CH=CH), 6.81 (3H, br s, OH, NH, NH_2_), 3.67-3.66 (2H, m, NH-CH_2_CH_2_), 3.52-3.50 (2H, m, CH_2_-OH), 3.43-3.41 (2H, m, NH-CH_2_), 3.12-3.11 (2H, m, NH-CH_2_CH_2_), 2.05-2.01 (2H, m, NH-CH_2_CH_2_); ^13^C-NMR (125 MHz, DMSO-*d*_6_) δ ppm 165.7, 165.4, 165.3, 159.0, 145.0, 144.9, 144.1, 143.8, 141.6, 133.4, 131.4, 130.2, 129.9, 129.8, 129.2, 127.5, 126.9, 126.4, 124.8, 57.6, 44.8, 39.7, 35.2, 32.1; IR ν_max_ (cm^−1^) 3378, 3267 (OH, NH, NH_2_), 3078, 3056, 3034 (CH_arom_), 2934, 2865 (CH_aliph_), 1717 (CH=CH), 1654, 1570 (C=C, C=N), 1343 (SO_2_NH_2_), 1256 (C-OH_phenol_), 1156 (SO_2_NH_2_), 1037 (C-OH_aliph_).

*(E)-4-{[(4-[(2-hydroxyethyl)amino]-6-{[4-(3-hydroxystyryl)phenyl]amino}-1,3,5-triazin-2-yl)amino]methyl}benzenesulfonamide* (**28**): 29.3%; vanilla solid; mp 138–140 °C; ^1^H-NMR (500 MHz, DMSO-d_6_) δ ppm 9.17 (4H, br s, NH, NH_2_), 7.79 (2H, d, *J* = 7.8 Hz, CH), 7.71 (2H, d, *J* = 7.8 Hz, CH), 7.64-7.63 (1H, m, CH), 7.58-7.56 (3H, m, CH), 7.47 (2H, d, *J* = 8.5 Hz, CH), 7.38 (2H, d, *J* = 8.5 Hz, CH), 6.97 (3H, br s, OH, NH, NH_2_), 6.85 (1H, d, *J* = 16.1 Hz, CH=CH), 6.83 (1H, d, *J* = 16.1 Hz, CH=CH), 4.72 (2H, s, NH-CH_2_), 3.62-3.59 (4H, m, NH-CH_2_, CH_2_-OH); ^13^C-NMR (125 MHz, DMSO-*d*_6_) δ ppm 165.9, 165.7, 165.4, 159.3, 145.2, 144.7, 144.6, 144.5, 143.2, 135.1, 132.3, 131.6, 131.1, 129.2, 128.9, 126.8, 125.9, 124.3, 117.6, 56.4, 43.5, 40.1; IR ν_max_ (cm^−1^) 3464, 3378 (OH, NH, NH_2_), 3074, 3055, 3037 (CH), 2991, 2962 (CH_aliph_), 1716 (CH=CH), 1653, 1589, 1574 (C=C, C=N), 1334 (SO_2_NH_2_), 1260 (C-OH_phenol_), 1156 (SO_2_NH_2_), 1026 (C-OH_aliph_).

*(E)-4-{2-[(4-[(2-hydroxyethyl)amino]-6-{[4-(4-hydroxystyryl)phenyl]amino}-1,3,5-triazin-2-yl)amino]ethyl}benzenesulfonamide* (**29**): 65.7%; brown solid; mp 73–75 °C; ^1^H-NMR (500 MHz, DMSO-d_6_) δ ppm 9.14 (4H, br s, NH, NH_2_), 7.76 (2H, d, *J* = 7.8 Hz, CH), 7.65 (2H, d, *J* = 8.0 Hz, CH), 7.35 (2H, d, *J* = 8.0 Hz, CH), 7.12 (2H, d, *J* = 8.0 Hz, CH), 7.03 (2H, d, *J* = 7.8 Hz, CH), 6.89 (2H, d, *J* = 8.0 Hz, CH), 6.71 (1H, d, *J* = 16.1 Hz, CH=CH), 6.68 (1H, d, *J* = 16.1 Hz, CH=CH), 6.61 (3H, br s, OH, NH, NH_2_), 3.78 (2H, t, *J* = 7.8 Hz, NH-CH_2_CH_2_), 3.56-3.51 (4H, m, CH_2_, CH_2_-OH) 2.89 (2H, t, *J* = 7.8 Hz, NH-CH_2_CH_2_); ^13^C-NMR (125 MHz, DMSO-*d*_6_) δ ppm 166.9, 166.6, 166.0, 157.0, 144.2, 142.4, 141.6, 139.2, 138.7, 131.0, 131.1, 130.0, 129.2, 127.2, 126.4, 121.6, 116.6, 61.2, 43.4, 43.2, 34.7; IR ν_max_ (cm^−1^) 3421, 3364 (OH, NH, NH_2_), 3077, 3054, 3021 (CH_arom_), 2962, 2928 (CH_aliph_), 1718 (CH=CH), 1658, 1582, 1560 (C=C, C=N), 1333 (SO_2_NH_2_), 1252 (C-OH_phenol_), 1154 (SO_2_NH_2_), 1029 (C-OH_aliph_).

*(E)-4-{2-[(4-[(4-hydroxyphenyl)amino]-6-{[4-(2-hydroxystyryl)phenyl]amino}-1,3,5-triazin-2-yl)amino]ethyl}benzenesulfonamide* (**30**): 49.5%; light brown solid; mp 147–149 °C; ^1^H-NMR (500 MHz, DMSO-d_6_) δ ppm 9.21 (4H, br s, NH, NH_2_), 7.81(2H, d, *J* = 7.8 Hz, CH), 7.79 (2H, d, *J* = 7.8 Hz, CH), 7.64-7.59 (4H, m, CH), 7.51 (2H, d, *J* = 8.0 Hz, CH), 7.30 (2H, d, *J* = 8.0 Hz, CH), 7.19 (2H, d, *J* = 8.0 Hz, CH), 6.75 (2H, d, *J* = 8.0 Hz, CH), 6.71 (3H, br s, OH, NH, NH_2_), 6.67-6.64 (2H, m, CH=CH), 3.79 (2H, t, *J* = 7.8 Hz, NH-CH_2_CH_2_), 2.97 (2H, t, *J* = 7.8 Hz, NH-CH_2_CH_2_); ^13^C-NMR (125 MHz, DMSO-*d*_6_) δ ppm 166.9, 166.3, 162.9, 157.8, 156.9, 143.8, 142.4, 142.0, 139.7, 134.8, 134.2, 134.0, 132.5, 132.1, 129.6, 129.2, 128.6, 127.1, 124.2, 122.7, 122.5, 119.8, 116.9, 115.8, 43.1, 34.8; IR ν_max_ (cm^−1^) 3327, 3234 (OH, NH, NH_2_), 3055, 3036 (CH_arom_), 2991 (CH_aliph_), 1677 (CH=CH), 1590, 1576 (C=C, C=N), 1332 (SO_2_NH_2_), 1264 (C-OH_phenol_), 1182 (SO_2_NH_2_).

*(E)-4-{2-[(4-[(4-hydroxyphenyl)amino]-6-{[4-(3-hydroxystyryl)phenyl]amino}-1,3,5-triazin-2-yl)amino]ethyl}benzenesulfonamide* (**31**): 26.3%; vanilla solid; mp 151–152 °C; ^1^H-NMR (500 MHz, DMSO-d_6_) δ ppm 9.16 (4H, br s, NH, NH_2_), 7.79 (2H, d, *J* = 7.8 Hz, CH), 7.72 (2H, d, *J* = 7.8 Hz, CH), 7.37 (2H, d, *J* = 8.1 Hz, CH), 7.30-7.25 (2H, m, CH), 7.12 (2H, d, *J* = 8.1 Hz, CH), 7.07-7.02 (2H, m, CH), 6.99 (2H, d, *J* = 8.0 Hz, CH), 6.71 (2H, d, *J* = 8.0 Hz, CH), 6.69-6.66 (2H, m, CH=CH), 6.61 (3H, br s, OH, NH, NH_2_), 3.72 (2H, t, *J* = 7.8 Hz, NH-CH_2_CH_2_), 2.90 (2H, t, *J* = 7.8 Hz, NH-CH_2_CH_2_); ^13^C-NMR (125 MHz, DMSO-*d*_6_) δ ppm 166.9, 166.7, 166.5, 158.4, 156.9, 143.9, 142.1, 139.2, 137.8, 133.5, 131.3, 129.9, 129.7, 129.6, 129.3, 129.2, 128.9, 126.9, 123.5, 121.4, 119.3, 116.9, 116.0, 113.2, 43.3, 34.5; IR ν_max_ (cm^−1^) 3324, 3218 (OH, NH, NH_2_), 3075, 3056, 3037 (CH_arom_), 3020, 2991, 2964 (CH_aliph_), 1681 (CH=CH), 1590, 1576, 1506 (C=C, C=N), 1332 (SO_2_NH_2_), 1262 (C-OH_phenol_), 1182 (SO_2_NH_2_).

*(E)-4-{2-[(4-[(4-hydroxyphenyl)amino]-6-{[4-(4-hydroxystyryl)phenyl]amino}-1,3,5-triazin-2-yl)amino]ethyl}benzenesulfonamide* (**32**): 53.1%; brown solid; mp 123–124 °C; ^1^H-NMR (500 MHz, DMSO-d_6_) δ ppm 9.14 (4H, br s, NH, NH_2_), 6.98 (2H, d, *J* = 7.8 Hz, CH), 6.95 (2H, d, *J* = 7.8 Hz, CH), 7.41 (2H, d, *J* = 8.1 Hz, CH), 7.26 (2H, d, *J* = 8.0 Hz, CH), 7.18 (2H, d, *J* = 8.1 Hz, CH), 7.16 (2H, d, *J* = 8.0 Hz, CH), 7.04 (2H, d, *J* = 8.0 Hz, CH), 7.00 (2H, d, *J* = 8.0 Hz, CH), 6.86 (1H, d, *J* = 16.1 Hz, CH=CH), 6.84 (1H, d, *J* = 16.1 Hz, CH=CH), 6.65 (3H, br s, OH, NH, NH_2_), 3.68 (2H, t, *J* = 7.4 Hz, NH-CH_2_CH_2_), 2.83 (2H, t, *J* = 7.4 Hz, NH-CH_2_CH_2_); ^13^C-NMR (125 MHz, DMSO-*d*_6_) δ ppm 166.8, 166.3, 166.1, 158.1, 157.2, 143.2, 142.9, 142.8, 139.4, 134.7, 132.4, 128.7, 128.2, 128.0, 127.6, 127.1, 126.8, 126.4, 124.7, 121.2, 199.8, 42.8, 34.1; IR ν_max_ (cm^−1^) 3328 (OH, NH, NH_2_), 3075, 3055, 3036 (CH_arom_), 2990, 2934 (CH_aliph_), 1721 (CH=CH), 1588, 1576 (C=C, C=N), 1332 (SO_2_NH_2_), 1262 (C-OH_phenol_), 1181 (SO_2_NH_2_).

### 4.3. Determination of Antioxidant Activity by ABTS Method

BioTekTM CytationTM 3 Cell Imaging Multi-Mode Reader (BioTek, Winooski, VT, USA) equipment was used for measurement.

A stock solution containing free cation radicals ABTS**^.^**^+^ was prepared as follows: 10 mL of an aqueous solution of ABTS (7.4 mmol) and 10 mL of an aqueous solution of potassium peroxodisulfate (2.6 mmol) were mixed and allowed to stand at room temperature in the dark for 24 h. After the end of the incubation period, a 50 mL volumetric flask was charged with 1.1 mL of ABTS stock solution and filled up with ethanol. This solution of ABTS**^.^**^+^ was used for the determination of antioxidant activity.

Tested compounds were dissolved in DMSO. Solutions of concentration 1 × 10^−2^ M, 1 × 10^−3^ M, 5 × 10^−4^ M, 1 × 10^−4^ M, 5 × 10^−5^ M, 1 × 10^−5^ M, and 1 × 10^−6^ M were prepared.

Antioxidant activity of tested compounds was determined as follows: 200 μl of a solution of ABTS**^.^**^+^ and 10 μl of a solution of the tested compound of appropriate concentration were mixed. Antioxidant activity was determined by measurement of absorbance at 754 nm (max absorbance of ABTS**^.^**^+^) after 0, 5, 30, and 60 min. Pure DMSO was used as a blank. Trolox and ascorbic acid served as standards (solutions were prepared at the same concentrations as tested compounds).

ABTS cation radical scavenging ability of tested compounds expressed as %inhibition was calculated using the following equation [30]:

(1) $$\%=\frac{A_{B}-A}{A_{B}}\;100,$$

where *A_B_* means absorbance of blank; *A* means absorbance of the sample.

The values of EC_50_ were determined as follows: The values of %inhibition of ABTS**^.^**^+^ radical at 0, 5, 30, and 60 min at concentrations of 1 × 10^−2^ M, 1 × 10^−3^ M, 5 × 10^−4^ M, 1 × 10^−4^ M, 5 × 10^−5^ M, 1 × 10^−5^ M, and 1 × 10^−6^ M were determined; a plot of concentration versus %inhibition was obtained from the data; a number 50 was substituted for the unknown **x** (50% inhibition) in the trendline equation, and value EC_50_ was calculated.

All calculated results are shown in Table 1.

## Figures and Tables

**Table 1:** ABTS cation radical scavenging ability of tested compounds expressed as %inhibition at different times (0, 5, 30, and 60 min) and different concentrations (1 × 10^−2^ M and 1 × 10^−4^ M); ABTS cation radical scavenging ability of the most active compounds expressed as EC_50_ (μM) at 60 min.

Compound	n	R_1_	R_2_	1 × 10^−2^ M; (%) ^2^				1 × 10^−4^ M; (%) ^2^				EC_50_ (μM) ^2^
				0 min	5 min	30 min	60 min	0 min	5 min	30 min	60 min	60 min
**3**	1	NH-CH_2_CH_2_OH	Cl	45.07	56.11	64.33	72.56	31.56	39.88	45.67	58.34	136.56
**4**	2	NH-CH_2_CH_2_OH	Cl	49.34	61.78	76.45	80.89	32.59	43.27	46.78	52.47	128.37
**5** ^1^	1	NH-C_6_H_4_(1,4)-4-OH	Cl	92.05	92.00	91.88	91.88	31.52	38.51	44.71	73.51	26.59
**6** ^1^	2	NH-C_6_H_4_(1,4)-4-OH	Cl	91.38	91.26	91.21	91.21	45.38	49.84	63.65	73.44	17.16
**7** ^1^	1	NH-CH_2_-C_6_H_4_(1,4)-4-SO_2_NH_2_	NH-CH_2_CH_2_-C_6_H_4_(1,4)-4-SO_2_NH_2_	35.58	47.98	58.35	66.69	<30	<30	<30	35.57	887.86
**8** ^1^	2	NH-CH_2_-C_6_H_4_(1,4)-4-SO_2_NH_2_	NH-CH_2_CH_2_-C_6_H_4_(1,4)-4-SO_2_NH_2_	51.53	74.64	79.54	90.36	31.46	35.24	40.99	41.83	900.00
**9** ^1^	1	[N(CH_2_CH_2_)_2_N]COOCH_3_	NH-CH_2_CH(OH)CH_2_OH	90.70	92.84	92.95	92.90	<30	31.01	39.92	52.99	61.29
**10** ^1^	1	[N(CH_2_CH_2_)_2_N]CH_2_COOCH_3_	NH-CH_2_CH(OH)CH_2_OH	63.98	82.13	87.15	91.77	<30	35.07	34.73	50.06	75.47
**11** ^1^	1	[N(CH_2_CH_2_)_2_N]CH_2_COOCH_3_	NH-CH_2_CH_2_-C_6_H_4_(1,4)-4-SO_2_NH_2_	79.20	86.47	88.33	88.39	<30	34.28	42.90	64.38	43.84
**12** ^1^	1	[N(CH_2_CH_2_)_2_N]CH_2_CH_2_COOCH_3_	NH-C_6_H_4_(1,4)-4-OH	89.63	89.69	89.69	89.69	38.28	39.41	41.89	60.66	51.47
**13** ^1^	1	NH-C_6_H_4_(1,4)-4-OH	NH-CH_2_CH(OH)CH_2_OH	89.40	89.29	89.18	89.12	33.55	36.31	48.71	78.24	27.78
**14** ^1^	1	NH-CH_2_-C_6_H_4_(1,4)-4-SO_2_NH_2_	NH-CH_2_CH_2_CH_2_OH	43.81	62.80	75.54	82.19	<30	<30	30.67	49.16	138.21
**15**	1	NH-CH_2_CH_2_CH_2_OH	-NH-C_6_H_4_(1,4)-(C=O)-CH=CH-C_6_H_4_(1,4)-4-H	52.03	68.04	75.88	81.57	<30	<30	<30	42.62	229.01
**16**	2	NH-CH_2_CH_2_CH_2_OH	-NH-C_6_H_4_(1,4)-(C=O)-CH=CH-C_6_H_4_(1,4)-4-H	52.60	70.01	85.29	89.12	<30	<30	31.63	46.34	158.12
**17**	1	NH-CH_2_CH_2_CH_2_OH	-NH-C_6_H_4_(1,4)-(C=O)-CH=CH-C_6_H_4_(1,4)-3-OH	89.57	89.57	89.63	89.63	24.70	31.91	34.62	50.79	66.39
**18**	1	NH-CH_2_CH_2_CH_2_OH	-NH-C_6_H_4_(1,4)-(C=O)-CH=CH-C_6_H_4_(1,4)-2-OH	90.70	90.36	89.97	89.74	34.28	35.41	37.32	57.56	64.43
**19**	2	NH-CH_2_CH_2_CH_2_OH	-NH-C_6_H_4_(1,4)-(C=O)-CH=CH-C_6_H_4_(1,4)-2-OH	91.43	91.32	91.04	90.76	<30	<30	<30	40.03	170.44
**20**	1	NH-CH_2_CH_2_OH	-NH-C_6_H_4_(1,4)-(C=O)-CH=CH-C_6_H_4_(1,4)-2-OH	90.59	90.19	89.80	89.52	<30	<30	30.90	49.39	127.74
**21**	2	NH-CH_2_CH_2_OH	-NH-C_6_H_4_(1,4)-(C=O)-CH=CH-C_6_H_4_(1,4)-2-OH	90.08	89.40	88.84	89.07	<30	<30	<30	47.07	153.70
**22**	2	NH-CH_2_CH_2_OH	-NH-C_6_H_4_(1,4)-(C=O)-CH=CH-C_6_H_4_(1,4)-3-OH	89.12	89.01	89.01	89.12	25.49	34.79	47.81	65.28	20.16
**23**	2	NH-CH_2_CH(OH)CH_2_OH	-NH-C_6_H_4_(1,4)-(C=O)-CH=CH-C_6_H_4_(1,4)-2-OH	91.15	91.04	90.76	90.53	36.25	38.85	40.59	58.97	74.49
**24**	2	NH-C_6_H_4_(1,4)-4-OH	-NH-C_6_H_4_(1,4)-(C=O)-CH=CH-C_6_H_4_(1,4)-2-OH	89.40	87.32	88.56	90.19	37.72	39.47	42.28	69.34	45.78
**25**	2	[N(CH_2_CH_2_)_2_N]CH_2_CH_2_COOCH_3_	-NH-C_6_H_4_(1,4)-(C=O)-CH=CH-C_6_H_4_(1,4)-4-OH	90.47	91.83	91.77	92.11	40.03	46.96	55.25	87.09	17.22
**26**	1	NH-CH_2_CH_2_CH_2_OH	-NH-C_6_H_4_(1,4)-CH=CH-C_6_H_4_(1,4)-3-OH	55.92	65.05	74.47	82.92	<30	<30	<30	39.63	344.87
**27**	2	NH-CH_2_CH_2_CH_2_OH	-NH-C_6_H_4_(1,4)-CH=CH-C_6_H_4_(1,4)-3-OH	83.37	90.19	91.66	91.71	<30	<30	<30	43.19	264.71
**28**	1	NH-CH_2_CH_2_OH	-NH-C_6_H_4_(1,4)-CH=CH-C_6_H_4_(1,4)-2-OH	80.60	84.05	88.03	89.45	<30	<30	<30	<30	305.67
**29**	2	NH-CH_2_CH_2_OH	-NH-C_6_H_4_(1,4)-CH=CH-C_6_H_4_(1,4)-4-OH	79.20	85.91	88.33	88.39	<30	<30	<30	30.73	282.36
**30**	2	NH-C_6_H_4_(1,4)-4-OH	-NH-C_6_H_4_(1,4)-CH=CH-C_6_H_4_(1,4)-2-OH	71.03	75.09	81.96	87.83	<30	<30	<30	44.14	199.34
**31**	2	NH-C_6_H_4_(1,4)-4-OH	-NH-C_6_H_4_(1,4)-CH=CH-C_6_H_4_(1,4)-3-OH	90.08	89.91	89.80	89.57	28.53	31.97	34.28	48.26	79.34
**32**	2	NH-C_6_H_4_(1,4)-4-OH	-NH-C_6_H_4_(1,4)-CH=CH-C_6_H_4_(1,4)-4-OH	91.04	90.98	90.81	90.64	<30	<30	32.31	<30	289.51
**trolox**	-	-	-	89.29	89.23	88.18	89.23	34.96	35.41	37.61	41.49	178.33
**Ascorbic acid**	-	-	-	88.33	88.45	88.33	88.45	29.09	28.81	29.83	31.07	147.47

^1^ Compounds previously reported in [48]; ^2^ Errors in the range of 1–5% of the reported value (mean from three different assays).
